# Dynamic rheological properties of polyurethane-based magnetorheological gels studied using oscillation shear tests

**DOI:** 10.1039/c8ra10297j

**Published:** 2019-04-01

**Authors:** Guang Zhang, Huixing Wang, Jiong Wang, Jiajia Zheng, Qing Ouyang

**Affiliations:** School of Mechanical Engineering, Nanjing University of Science and Technology Nanjing 210094 China wjiongz@njust.edu.cn; School of Engineering, Zhejiang Normal University Zhejiang 321004 China; School of Mechanical Engineering, Jiaxing University Zhejiang 314001 China

## Abstract

This paper studies the dynamic rheological behaviors of magnetorheological (MR) gels containing different CIP weight fractions suspended in polyurethane (PU). The dynamic characteristics of prepared MR gels are obtained using large amplitude oscillation shearing (LAOS). The influence of strain amplitude, applied coil current, CIP weight fraction and driving angular frequency on the dynamic rheological properties of MR gels are systematic discussed. The results demonstrate the onset strain from linear viscoelastic region to non-linear viscoelastic region increases with applied coil current. The maximum loss modulus increases with applied coil current and CIP mass fraction. The larger the coil current and CIP mass fraction, the greater the shear strain corresponding to the maximum value. Microstructural variation of self-assembled copolymer chains and magnetic-induced CIP chains at different strain amplitudes, applied coil currents and CIP weight fractions are proposed as an explanation of the non-linear rheological behaviors of PU-based MR gels.

## Abstract 2

This paper studies the dynamic rheological behaviors of magnetorheological (MR) gels containing different CIP weight fractions suspended in polyurethane (PU).

## Graphical abstract

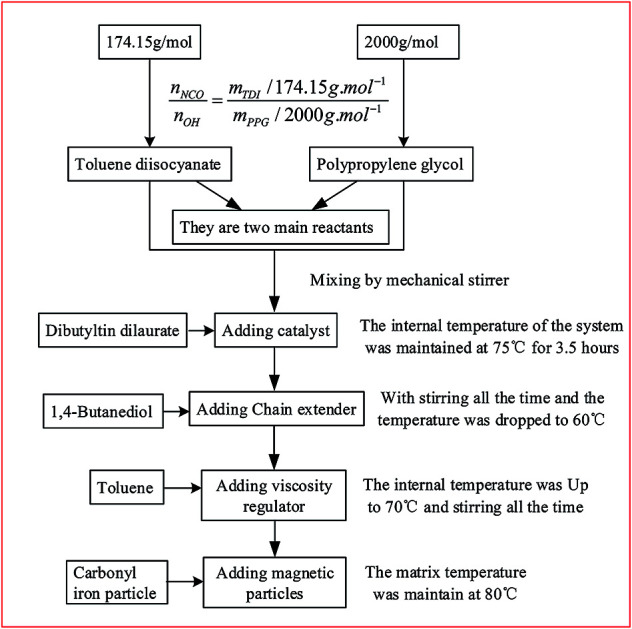

## 1 Introduction

MR gels are suspensions of magnetizable particles (*e.g.*, iron particles, carbonyl iron powder, *etc.*) in a viscoelastic carrier gel (*e.g.*, polyurethane, silicone, *etc.*). They are field-controllable materials, whose rheological properties can be dramatically altered by applying a magnetic field. In the past few years, the magneto-induced properties of MR gels have been studied.^1–3^ Conventional MR materials consist of MR fluids, MR foams and MR elastomers.^4^ The molecular chain length and rheological behavior of polymer gels can be controlled simply by changing the concentration and molar mass of reactants. Therefore, it has better temperature stability compared with MR elastomers.^5–8^

With those advantages, more and more researchers are interested in MR gels in the past decade. An *et al.* have recently self-developed swollen physical magneto-responsive gels with dispersing soft CIPs by using the method of self-assembly of triblock copolymers. Then the linear viscoelastic behaviors (LVEHs) of triblock MR gels containing soft magnetic particles have been reported.^9^ Xu *et al.* prepared a series of polyurethane (PU)-based MR gels with different weight fractions of carbonyl iron particles (CIPs) and the LVEPs of products were tested.^10^ Wang *et al.* investigated the dynamic mechanical behavior of magnetically responsive shear-stiffening gel by using a modified Split Hopkinson Bar (SHPB) system.^11^ At first it was taken for granted that the dynamic mechanical behaviors of MR gels would be similar to those of MR elastomers. Actually, this is not the case. Compared with MR elastomer, the relaxation of storage modulus and loss modulus for MR gels can be observed for several minutes under the applied of step magnetic field. A plausible explanation is that within MR gels system, soft magnetic have a certain degree of freedom in the base fluid, with the ability for local rearrangement of the original particle chains under the application of magnetic particles, which is similar to MR fluid. Internal particles will migrate locally and arrange into chains parallel to the magnetic field, which takes a certain time (relaxation phenomenon). The length of time depends on the hindrance ability of base carrier to particles.^12–14^ MR elastomers are totally different for particles in it are fixed in the matrix. Therefore, there is no relaxation phenomenon.^15^

The dynamic mechanical performance of MR materials is widely recognized to be related to the microstructures of the magnetic particles chains and rearrangement mechanism of the microstructures in response to an applied magnetic field.^9^ In MR fluids, the induced structures contribute to the observation of significant anisotropic properties. When compared to MR elastomers, however, the particles and particle strings within MR elastomers are regarded as “frozen in” because of the high viscoelastic forces coming from the embedded polymer chains.^5^ In a word, when applied magnetic field, different from MR fluid only with particle strings chains, however, within MR gels and MR elastomers not only include particle strings chains, but also include natural molecular chain chains. In particular, our interest in MR gels comes from particles mobility in system and how to move, which might result in a strong dynamic non-linear behavior associated. The issue is crucial to capture some of the special behavior of MR gels such the rheological response with stepwise magnetic field.^16^

Storage modulus and loss modulus are the most frequently used to characterize dynamic properties and can be comfortable defined in the LVE regime but that is not sufficient to systematic capture the magnetic-induced dynamic mechanical properties studied here. Stepping out of the LVE regime and entering the range of activities in non-linear viscoelasticity (NLVE) indicates the application of techniques like LAOS that has already been widely used in various MR fluids-based and MR elastomer-based devices.^6,17,18^ The engineering-based importance of LAOS in MR technology is transparent since many applications work under high strain amplitude dynamic loading conditions. From ref. 10, it can be found that when the dynamic properties change dramatically with increasing strain amplitude, then strain-induced nonlinearity is generated. The strain dependent behavior of the dynamic characteristics of MR materials is called Payne effect. The nonlinear rheological properties of MR fluids and MR elastomers have been widely studied,^19–22^ whilst the rheological properties of MR gel, as new material, hasn't fully investigated.

In this work, we carry out LAOS^23^ (0.001–100%) to study PU-based MR gels with different weight fractions. The magnetic-induced CIPs chains still has local rearrange ability with certain degree of freedom under the action of coil current, which is different from the fixed situation of particles in MR elastomers. The LAOS testing technique gives an ordinary way to catch the transformation process of nonlinearity. It is worth to note there that the storage and loss modulus measured by commercial rheometer is the first harmonic modulus. In this paper, the applied coil current and CIP weight fraction dependent rheological behaviors of PU-based MR gels were investigated. The MR gels with different CIP mass fraction, *i.e.* 0%, 40%, 50%, 60%, 70% and 80% are firstly developed and the physical characterizations of MR gels, such as magnetic properties, size, shape and interfacial wettability between CIPs and the PU matrix were measured and discussed accordingly. Then the dynamic properties of prepared MR gels are obtained under LAOS. Finally, the influences of stain amplitude, driving angular frequency and applied coil current on the dynamic non-linearity characteristics of MR gels were systematic discussed.

## 2 Experimental section

### 2.1 Raw materials

Table 1 showing the raw materials and its producer for preparing MR gels. There are six raw materials, *i.e.* toluene diisocyanate and polypropylene glycol was used as two main reactants, 1,4-butanediol was used as chain extender, dibutyltin dilaurate was used as catalyst, toluene was used as viscosity regulator and carbonyl iron particle was used as magnetic material.

**Table 1:** The raw materials and its producer for preparing MR gels

Raw materials	Producer
Toluene diisocyanate (TDI; 2,4- ≈80%, 2,6- ≈20%)	Yantai Wanhua Polyurethanes Co., Ltd, China
Polypropylene glycol (PPG-2000)	Sinopharm Chemical Reagent Co., Ltd, China
1,4-Butanediol (BDO)	The Third Petrochemical Factory, Tianjin Petrochemical Inc, China
Dibutyltin dilaurate (DBTDL)	Yantai Wanhua Polyurethanes Co., Ltd, China
Toluene	The Third Petrochemical Factory, Tianjin Petrochemical Inc, China
Carbonyl iron particle (type: CIP–CN)	Produced by BASF, Germany with an average particle size of about 3 μm

### 2.2 Preparation of MR gels

**Fig. 1:**
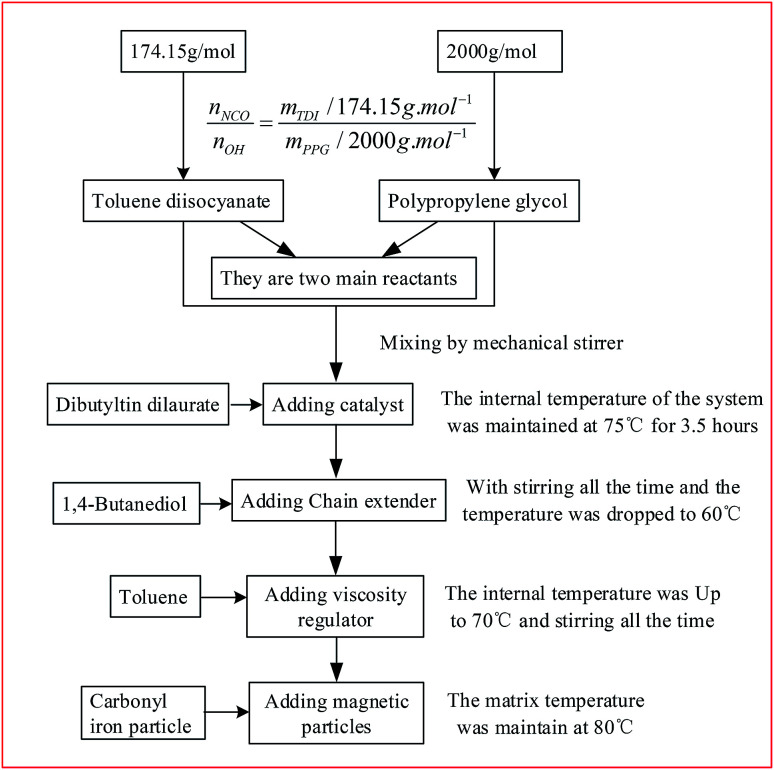
Preparation process of PU-based MR gels.

**Fig. 2:**
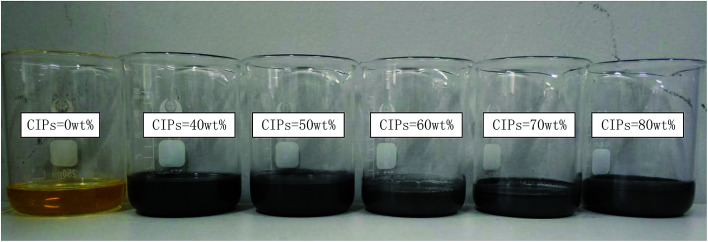
PU-based MR gels with different CIPs mass fraction.

The diagram of preparation process of PU-based MR gels is illustrated in Fig. 1.^8,10,24–27^ PU is chosen as matrix since it is a well-known polymer matrix for gel application with variable physical and mechanical properties.^28^ The mass fraction of CIPs of those products were choose as 0, 40, 50, 60, 70 and 80%, respectively. Therefore, the six kinds of homemade products were named as sample 1, sample 2, sample 3, sample 4, sample 5 and sample 6, respectively. The photograph of the samples is shown in Fig. 2. The weight fraction of particles and matrix for six samples are shown in Table 2.

**Table 2:** Compositions of MR gels

Samples	CIP (wt%)	PU matrix (wt%)
Sample 1	0	100
Sample 2	40	60
Sample 3	50	50
Sample 4	60	40
Sample 5	70	30
Sample 6	80	20

### 2.3 Characterization and MR measurement

Scanning electron microscope (SEM) (Model Quant 250FEG) and vibrating sample magnetometer (VSM, Lake Shore 7407, USA) were used to study the morphology of CIPs and magnetic properties of MR gels, respectively. Dynamic properties measurements were carried out using a rotational rheometer (Anton Paar MCR physica 302) with a parallel-plate geometry in strain controlled mode and a magnetic field generator. The distance between two parallel-plates with a diameter of 20 mm was fixed at 1 mm throughout the tests. Magnetic field generator (MRD 180) can generate a uniform magnetic flux density in the gap from 0 to 480 mT by regulating the coil current from 0 to 2 A. The direction of the magnetic field is perpendicular to the shear direction during the dynamic behavior tests. In this study, oscillation tests were used to study the dynamic non-linear rheological properties. Under oscillatory test environment, the strain amplitude sweep test and the frequency sweep test were conducted at five kinds of current intensity, *i.e.* 0 A, 0.5 A, 1 A, 1.5 A and 2 A. In strain amplitude test, the strain amplitude range from 0.001% to 100% (LAOS) at fixed angular frequency *w* = 5 rad s^−1^. In the frequency sweep test, the angular frequency varied from 1 rad s^−1^ to 100 rad s^−1^ at a fixed strain amplitude *γ* = 0.005% to obtain the dynamic characteristics of MR gels. To maintain the consistency of each measurement, the interval between each test point is set to 10 seconds. In addition, all of the experiments were performed three times and then calculated average values to ensure the reproducibility of the obtained data. The above mentioned testing environmental temperature was kept at 25 °C.

## 3 Results and discussion

### 3.1 Material characteristics

**Fig. 3:**
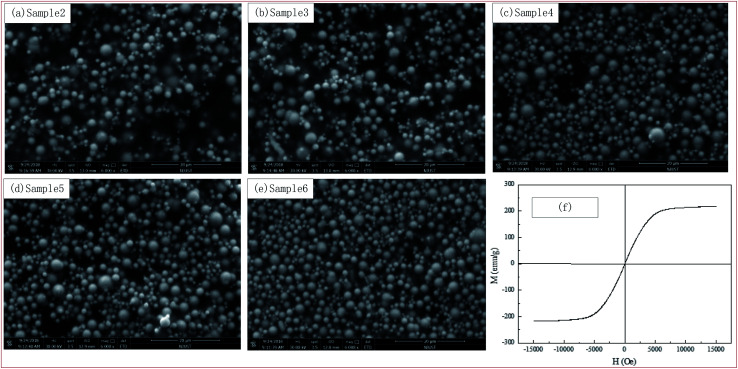
The microstructure of the PU-based MR gels observed by SEM and magnetic hysteresis loops properties of the CIPs.

The microstructure of developed MR gels were directly observed by SEM without applied magnetic field. The white points in Fig. 3(a)–(e) stand for the position of the CIPs and the dark background is the PU matrix. It can be seen from Fig. 3(a)–(e) that the CIPs were dispersed homogeneous in PU-based matrix and the higher the CIPs content, the denser the CIPs distribution. It proves the freshly prepared MR gels are actually isotropic from the distribution. In addition, it can be seen from Fig. 3(a)–(e) that the PU matrix is attached on the surface of CIPs and occupy the interspaces between it. It can be observed from the scale size in the Fig. 3(a)–(e) that the CIPs used are spherical and their diameter are concentrated range from 2 to 6 μm.

**Fig. 4:**
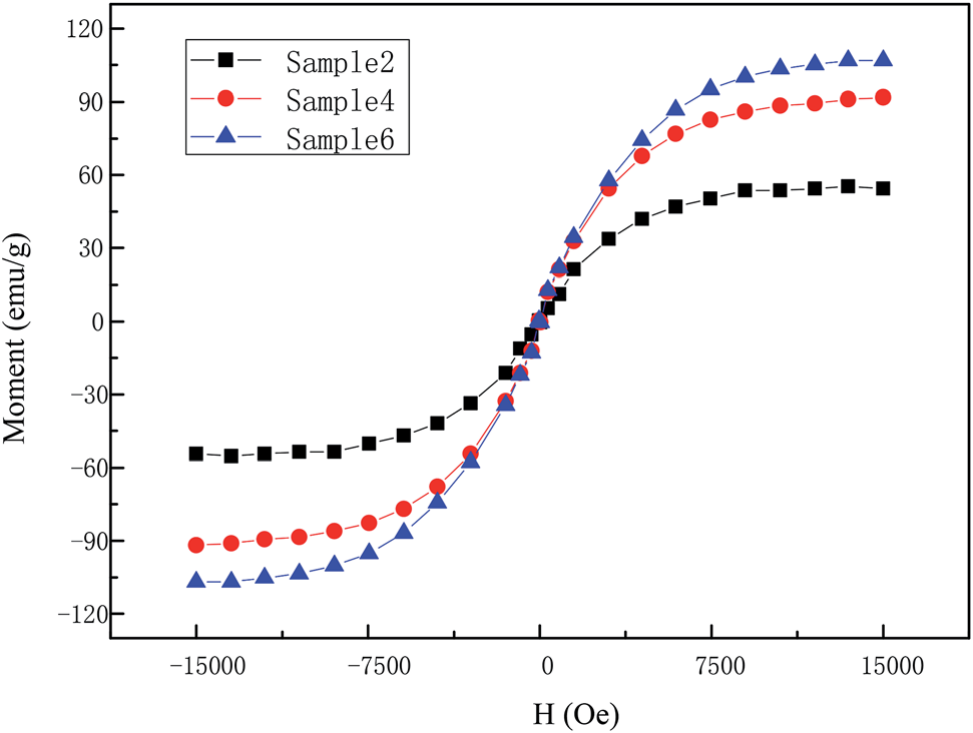
The magnetic hysteresis loops properties of partial freshly prepared MR gels.

The magnetic characteristics of CIPs and partial self-prepared MR gels, *i.e.* sample 2, sample 4 and sample 6 were studied using VSM with an applied magnetic field range of −15 to 15 kOe at room temperature (25 °C). As can be seen from Fig. 3(f) that CIPs can be regarded as a soft magnetic material for virtually no hysteresis. Combined with Fig. 3(f) and 4, it can be seen that they have the same magnetic properties: moment increases actually linearly along with magnetic field first and then tend to a saturation values. In addition, the saturation values of materials increase along with CIPs weight content. Take the first quadrant as an example, the saturation values were 211 emu g^−1^ for CIPs, 54 emu g^−1^ for sample 2, 91 emu g^−1^ for sample 4 and 106 emu g^−1^ for sample 6, respectively.

### 3.2 The influence of the magnetic field

**Fig. 5:**
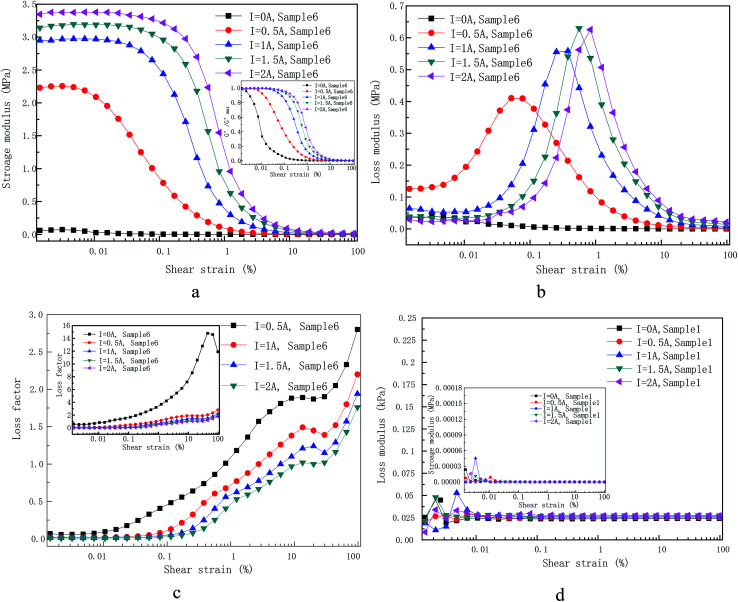
(a) Storage modulus, (b) loss modulus, (c) loss factor and (d) storage and loss modulus of pure PU matrix on the applied LAOS, gained at different applied currents. The normalized storage modulus is also shown in the inset of (a).

Fig. 5 was shown the storage modulus (a) and loss modulus (b) of self-made sample 6 under various applied coil currents as a function of the shear strain amplitude at a fixed angular frequency (5 rad s^−1^) and other samples have the same trend as sample 6. Various applied currents, *i.e.* 0 A, 0.5 A, 1 A, 1.5 A and 2 A, are shown. The loss factor (tan *δ* = *G*′′/*G*′) is also displayed in Fig. 5(c).

**Fig. 6:**
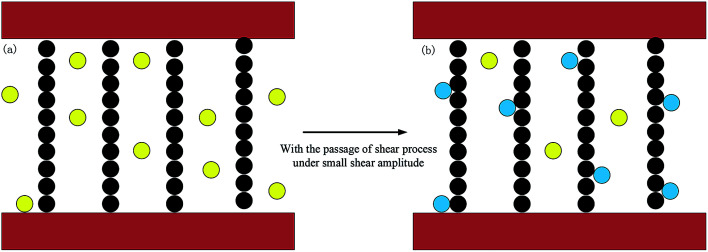
Micro-structure evolution under small shear amplitude. (The black circles represent the chained CIPs, the yellow circles represent the free CIPs and the blue circles represent the CIPs attracted by the chains.)

It can be observed from Fig. 5(a) that the storage modulus of sample 6 under different applied currents demonstrates a small increase at first. An *et al.* have reported that the time-dependence of the storage and loss modulus within low strain amplitude oscillatory shear when a step magnetic field is applied. The results of this work shown that the change of storage modulus for MR gels with step magnetic field has a relaxation property, that is, it continues to increases and then reach a stable value. It is recommended that quiescent optimal rearrangement of the particle chains in the small scales are reasonable responsible for the slowly evolution of the relaxation phenomenon.^29^ This is also a plausible explanation for a small increase at initial stage. Under the condition of small amplitude, the internal particle chain structure is basically finished once the coil current is applied. However, there are still some free CIPs due to the hindrance of the PU polymer chain structure as shown in Fig. 6(a), which is different from MR fluid.^30^ The free CIPs are attracted by the chain structure over the shear process under small strain amplitude, which forming a local strengthening effect like Fig. 8(b). Therefore, there is a slight increase in the storage modulus of materials under small strain amplitude.

In addition, it can be concluded from the storage modulus information in Fig. 5(a) that sample 6 approximately behaves as linear viscoelastic MR gel when there is no external magnetic flux density (*I* = 0 A). The indistinct Payne effect,^31^*i.e.* the storage modulus reduce slightly, was observed after shear strain amplitude at 0.003%. Under the action of magnetic flux density (*I* = 0.5 A, 1 A, 1.5 A and 2 A), the storage modulus is actually much large than in the absence of current and it increases with the increase of applied currents. The increase of storage modulus is result from the fact that the soft magnetic particles was magnetized to form dipoles at present of magnetic flux density, which are attracted by each other.^32^ The magneto-induced storage modulus displays an increasing trend along with coil current under the amplitude sweep until it rendezvous after loading strain at 30%. The storage modulus in the presence of applied coil current exhibits three characteristic areas: firstly, a relatively narrow LVE area up to a critical value related to applied coil current, *i.e.* 0.04% for 2 A, 0.03% for 1.5 A, 0.02% for 1 A and 0.006% for 0.5 A; secondly, there is a long decreasing tail along with shear strain amplitude and finally rendezvous together. As exhibited in the Fig. 5(a) (inset), the storage modulus values for different applied current is divided by the maximum storage modulus within the LVE area to highlight the non-linear variation of MR gels with shear strain amplitude. It can be observed that the transition value of strain amplitude increases along with applied currents.

There two kinds of chains in the MR gels system, *i.e.* polymeric chains formed by polymerization based on molar mass (matrix owned) and magnetic-induced CIPs chains (CIPs owned) after applied coil current according to CIPs concentration of MR gels. It can be concluded from Fig. 5(d) that the storage and loss modulus of the PU matrix does not vary during the whole shear strain from 0.001% to 100% and show current-independent characteristics. It is worth to note that loss modulus is much larger than storage modulus of PU matrix. Therefore, polymer chains behave like a linear sticky kettle with a broad LVE area since the loss modulus is much larger than storage modulus. The chains formed by the mutual attraction of polarized dipoles are very sensitive to external strain amplitude loading. This is because the larger the distance between dipoles, the smaller the force between them, which is the main reason for a cabined LVE area. The reduce of storage modulus or loss modulus can be interpreted in analogize to the Payne effect where the chains of the CIPs are separated due to strain or glassy bridges which link neighbouring CIPs is ruptured. In a word, the increasing shear strain signify a reduce in CIP–CIP interaction result from the increase in the distance between the CIPs centroid.

It has to be mentioned that the CIP–CIP distance become extremely complicated under the action of applied current after the rupture of the CIP chains and strings. In the absence of current, the inter-particle distance formed by external strain amplitude can be considered as evenly distributed, as shown in Fig. 3, and have good interfacial wettability between CIPs and the prepared PU matrix. Therefore, the macro-structure strain is shared by the inter-particle distances in a linear way as shown in Fig. 5(a) and (b). In the presence of a current, where chains and strings of CIPs are formed in PU-based matrix with a certain alignment, it can be expected that the inter-particle distance can be deemed as uniformly distributed only at very small strain value, *i.e.* 0.04% at 2 A, 0.03% at 1.5 A, 0.02% at 1 A and 0.006% at 0.5 A. However, for larger shear strain value beyond LVE point mentioned above, the CIP–CIP distance distribution, transforming into spatial distribution of interaction forces between polarized dipoles, which will make the dynamic rheological properties of MR gels completely differently. Higher strain amplitude will destroy the complete chains and strings formed before and then locally rearrange. Therefore, for the shear strain amplitude out of the LVE area, inter-particle distances in the shared chains are no longer uniform distributed under the magnetic environment and the CIPs tend to recombine owing to the dipole–dipole interaction force. Therefore, the strain amplitude of nonlinear initial strain can be regard as the strain required for destroy of the micro-structure of MR gels.

Loss modulus is a measure of information on energy dissipation,^33^ which is displayed in Fig. 5(b). The loss modulus for the absence of the current is practically approximate linear variation with shear strain. However, it become more complicated in the presence of the current. Diverse phenomenon from different shear strain area are summarized below: (1) after a smaller current (*I* = 0.5) is immediately applied, there is a strong jump in loss modulus at low strain compared with the loss modulus for the absence of current. Further increase the coil current, the loss modulus at low strain decreases with the increase of the coil current. (2) After the coil is energized, further increase the shear strain, the loss modulus increases rapidly with strain until a maximum value and then it decreases rapidly with the increase of strain. (3) The larger the coil current, the greater the shear strain corresponding to the maximum value. (4) The larger the coil current, the greater the maximum value. (5) Such a situation that an intersection of loss modulus for both samples may appear at higher strain amplitude.

Explanation of the loss modulus is much more complicated. The strong jump in loss modulus at low strain after a smaller current (*I* = 0.5) is immediately applied compared with it for the absence of current is also initiated by the weak magnetized dipoles mutual interaction. Therefore, the applied shear amplitude exceeds the critical value, the chains and strings formed by CIPs start to destroy and no longer tend to follow the increasing shear.^34^ However, the self-assembled copolymer chains will still prone to follow the shear, which result to the strong friction between the two chains. If the applied current increases further, the magnetic dipole–dipole interaction becomes stronger and the yield stress result from shear amplitude under low strain has no ability to rupture the clusters and strings, which makes the two kinds of chains move together along the shear direction and eventually reduce friction between them. Further increases the acting shear amplitude and exceed the LVE region, the clusters and strings was seriously damaged, which indicate that the CIPs has lost its memory of its initial location. It cost energy to destroy of particles chains and strings, which leads to the sharply increase of loss modulus. The maximum loss modulus and corresponding strain amplitude of sample 6 under different applied coil current are shown in Table 3. Further increasing strain (*i.e.* 0.836% for 2 A, 0.562% for 1.5 A, 0.378% for 1 A and 0.077% for 0.5 A) signifies a decrease in CIP–CIP magnetic mutual attraction as the increase in CIP–CIP distance. Compared with the increase of loss modulus caused by the displacement of particles or discontinuous strings. This effect dominates the decreasing tendency of the loss modulus. The clusters and strings have a tendency to become coarser after further increase the coil current and the dipole–dipole interaction become stronger. There should be larger shear amplitude to rupture coarser CIP chain, which result to the maximum loss modulus value and the strain amplitude corresponding to the maximum modulus increase with the increase of coil current.

**Table 3:** The maximum loss modulus and corresponding strain amplitude of sample 6 under different applied coil current

Applied coil current	Maximum loss modulus	Corresponding strain amplitude
*I* = 2 A	0.630 MPa	0.836%
*I* = 1.5 A	0.626 MPa	0.562%
*I* = 1 A	0.558 MPa	0.378%
*I* = 0.5 A	0.409 MPa	0.077%
*I* = 0 A	—	—

The loss factor, which is defined as the ratio of loss modulus to storage modulus, which is shown in Fig. 5(c). Several different applied coil currents, *i.e.* 0 A, 0.5 A, 1 A, 1.5 A and 2 A, are shown there with roughly the same shapes of the curve. Three different areas are displayed (flat, up and drop) without the action of coil current. It is reported in ref. 34 that the increase of loss factor polymer matrix is attributed to strain hardening of polymer chains. When the strain amplitude is greater than 30%, the copolymer chains in straightened in the shear direction, which will weaken the blocking effect of copolymer chains on the CIPs and eventually leads to the decrease of the loss factor. Differently, there are four various domains of loss factor (flat, up, flat, up) presenting under the action of current. The initial flat area corresponding the extremely cabined LVE domain where storage modulus is independent of strain in the present of coil current. The onset strain for the first increasing trend increases with the increase of applied coil current. The larger the current is, the weaker the increasing trend of loss factor is, which indicates that the larger contribution of the magnetized particle chains.

### 3.3 The influence of CIP weight fraction

**Fig. 7:**
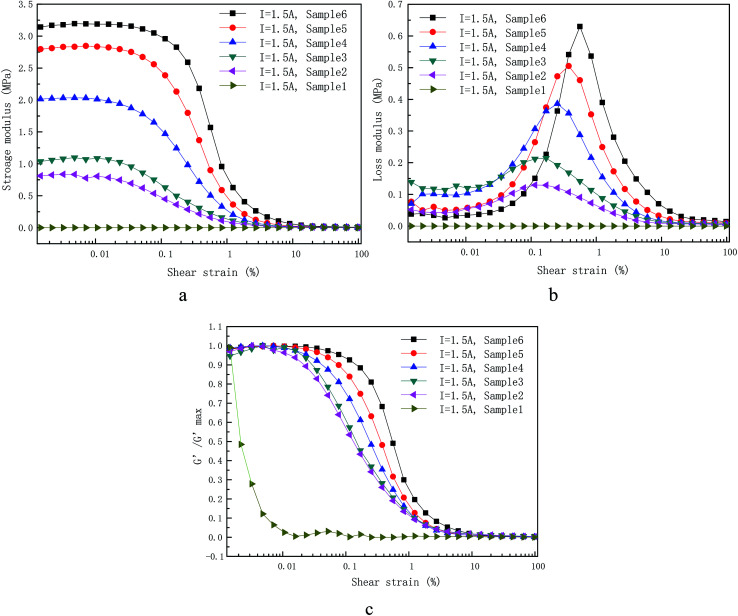
Dependence of (a) storage modulus, (b) loss modulus on the applied LAOS measured at 1.5 A with six different CIP weight concentration. The normalized storage modulus is also shown in the (c).

Fig. 7 exhibits the storage modulus (a) and loss modulus (b) of the as-prepared samples under constant applied coil current (*I* = 1.5 A) as a function of the shear strain amplitude at a fixed angular frequency (5 rad s^−1^) and other applied coil current have the same trend as current 1.5 A. The normalized storage modulus is also shown in the Fig. 7(c).

Storage modulus along with shear strain amplitude under the action of applied coil current with various weight fraction are shown in Fig. 7(a). The CIPs weight fraction was 0%, 40%, 50%, 60%, 70% and 80%. For the dynamic rheological properties, the storage modulus increases along with CIPs mass fraction as expected, which can be explained though Fig. 4 and ref. 35. Some obvious Payne effect was found after a confined LVE area with the critical strain values, *i.e.* 0.04% for sample 6, 0.02% for sample 5, 0.009% for sample 4 and so on. This phenomenon is defined here as the magnetic Payne effect that was used to distinguish the normal Payne effect in absence of applied coil current. In the other words, the critical strain value strongly decreases with CIPs weight fraction as shown in the Fig. 7(c). This can be interpreted that the dominant impact of magneto-induced CIP chains on the dynamic rheological properties for MR gels, compared with that of polymer chain, which enhance with increase of CIPs weight fraction.

**Fig. 8:**
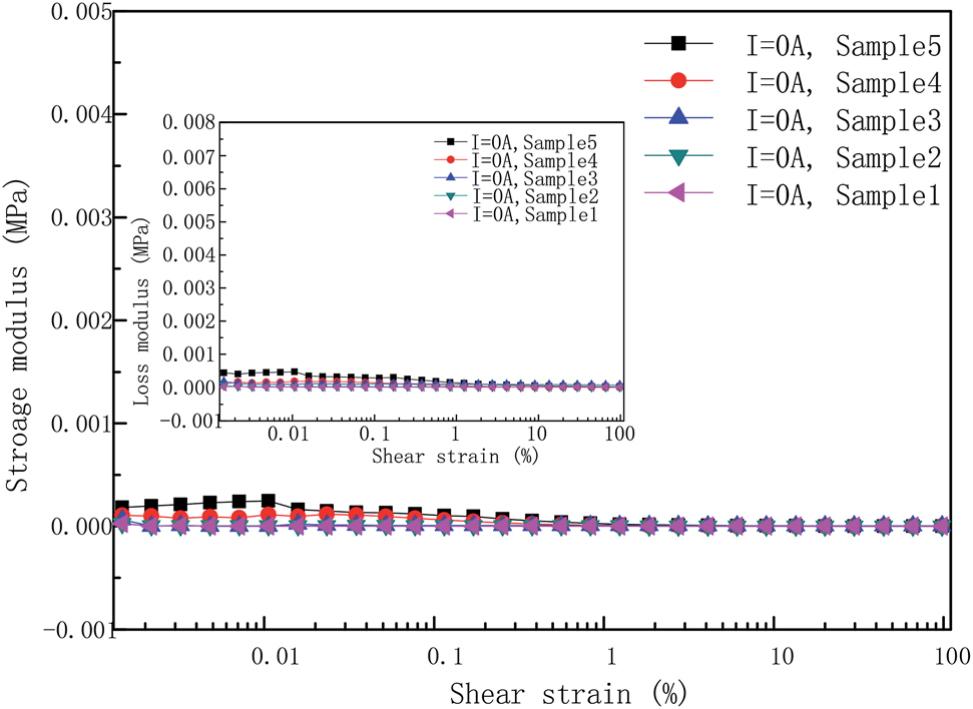
Dependence of storage and loss modulus on the applied OS measured at 0 A with five different CIP weight concentration.

As mentioned above, the rheological behavior of PU-based MR gels can be interpreted as the interaction of two chains (polymer chains and magnetic-induced CIPs chains). Rheological behavior in the absence of applied coil current is controlled by the PU copolymer chains. The contribution from the magnetic-induced chains can be ignored. For instance, the storage and loss modulus in absence of applied coil current only weakly depends on CIPs mass fraction as shown in Fig. 8. Differently, the dynamic rheological properties in the presence of applied coil current are mainly dominated by the magnetic-induced CIPs chains. The storage and loss modulus are determined by intensity of applied coil current and the CIPs weight fraction.

The relationship between loss modulus and strain amplitude of different CIPs weight fraction MR gels at constant applied coil current (*I* = 1.5 A) are shown in Fig. 7(b). The loss modulus of the MR gels with different CIPs weight fraction has the same trend with strain amplitude. The various trend of loss modulus with strain amplitude is similar to saddle shape. The plausible explanation for this trend of loss modulus for sample 6 under different applied coil current has been mentioned in the previous part. The loss modulus of samples with different CIPs mass fraction under the constant applied coil current has two obvious characteristics: (1) the strain amplitude corresponding to the maximum loss modulus increases with the increase of weight fraction, (2) the larger the coil current, the greater the maximum value. The maximum loss modulus and corresponding strain amplitude of samples with different CIP mass fraction at constant applied coil current (*I* = 1.5) are shown in Table 4.

**Table 4:** The maximum loss modulus and corresponding strain amplitude of samples with different mass fraction at constant applied coil current (*I* = 1.5 A)

Materials	Maximum loss modulus	Corresponding strain amplitude
Sample 6	0.626 MPa	0.562%
Sample 5	0.505 MPa	0.378%
Sample 4	0.386 MPa	0.254%
Sample 3	0.214 MPa	0.171%
Sample 2	0.129 MPa	0.115%
Sample 1	—	—

As known, the peak of loss modulus reflects the yielding process and has been related to the destruction of the clusters and strings structure of MR gel.^36^ The CIPs clusters and strings was destroyed under the condition of large strain amplitude and due to form a gap between CIPs. Then the local clusters absorb the free CIPs. The free CIPs overcome the resistance to approach the clusters. In this process, MR gel with higher weight fraction has relatively large amounts of free CIPs and therefore consumes a higher energy. In addition, MR gel with higher CIPs weight fraction can form thicker chains under applied coil current.^37^ The thicker the chain is, the larger the number of CIPs is, and the strain amplitude is divided equally by the CIPs in the chain. Thus, distance between CIPs caused by strain is smaller. Therefore, a greater strain amplitude is needed to reduce the interaction between CIPs so that the free CIPs can't be attracted. This is the reason that the strain corresponding to the maximum storage modulus increases with the increase of CIPs weight fraction.

**Fig. 9:**
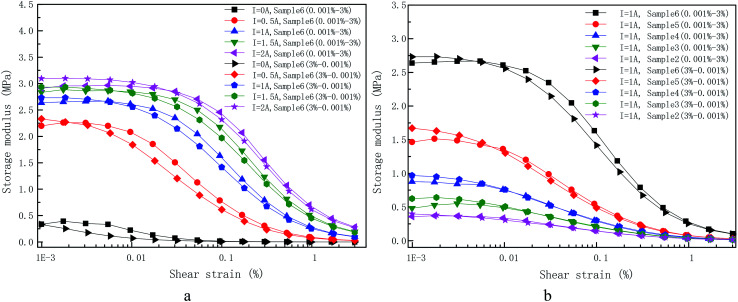
(a) Dependence of storage modulus of the sample 6 on strain amplitude (0.001–3–0.001%) measured at various applied coil current. (b) Dependence of storage modulus of samples with different CIPs weight fraction on strain amplitude (0.001–3–0.001%) under constant applied coil current (*I* = 1 A).

The reverse reversibility of magnetic-induced CIPs chains within prepared MR gels under larger shear strain amplitude (beyond the LVE region) is illustrated in Fig. 9. A complete circle strain amplitude sweep under shear mode was adopted to research the reverse reversibility at localized angular frequency at 5 rad s^−1^. The smaller angular frequency here was used to guarantee an enough long testing time but not exceed 10 min of the strain sweep. Stable quasi-static rearrangement of the magneto-induced dipole–dipole chains can be used to interpreted the continuous evolution of magneto-induced storage modulus. Ten minutes after applying the magnetic field, a stable micro-structure of the magnetic-induced CIPs chains has been formed. The time-dependence of storage and loss modulus after ten minutes can be ignored.^29^ Because of this reason, the initial shear strain amplitude sweep was started after ten minutes of the magnetization. After the strain amplitude range from 0.001% to 3% of the sample. The curve for the second shear strain amplitude sweep from 3% to 0.001% shows the same storage modulus when the strain amplitude beyond a critical value, *i.e.* 0.022% for 2 A, 0.0062% for 1.5 A, 0.005% for 1 A, 0.002% for 0.5 A and 0.001% for 0 A from Fig. 9(a), 0.006% for sample 6, 0.007% for sample 5, 0.01% for sample 4, 0.02% for sample 3 and 0.03% for sample 2 from Fig. 9(b). The strain amplitude corresponding to the transition point of storage modulus increases with the increase of applied coil current and decreases with the increase of CIPs mass fraction during the two cycles. The mechanism of this behavior is not yet clear and needs to further study. The general shape of the curve also remains the same. It appears that after the previous stable quiescent rearrangement (before the initial measurement), the magnetic-induced CIP chains rearrangement inside the MR gels is not effected by the inverse shear strain deformation even in the higher strain amplitude (non-LVE region).

**Fig. 10:**
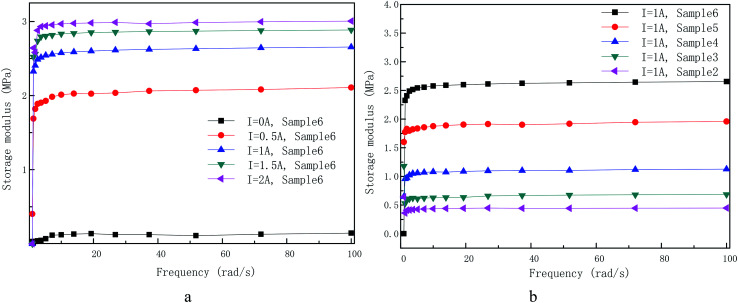
(a) Dependence of storage modulus of the sample 6 on frequency (1–100 rad s^−1^) measured at various applied coil current. (b) Dependence of storage modulus of samples with different CIPs weight fraction on frequency (1–100 rad s^−1^) under constant applied coil current (*I* = 1 A). The strain amplitude is keep at 0.005%.

Fig. 10 displays the change of storage modulus with frequency sweep for sample 6 at various applied coil current (a) and for samples with different CIPs weight fraction under constant applied coil current (b). They are tested in small strain of 0.005%. The frequency dependence of storage modulus was tested with and without action of coil current for sample 6 in Fig. 10(a) and (b), respectively. The result shows that the loading angular frequency has little impact on the dynamic rheological properties of self-developed MR gels, neither for the different applied coil current nor for the samples with different CIPs mass fraction. However, at low frequencies (<5 rad s^−1^), the storage modulus of self-developed MR gels increases slightly with the increase of shear frequency. This characteristic is advantageous to the control of MR devices and widens the application area of MR gels.

## 4 Conclusions

In this paper, MR gels with different CIPs weight fraction were prepared by mixing the CIPs and PU matrix using a mechanical stirrer. The microstructure of the PU-based MR gels observed by SEM shown that a homogeneous distribution of the CIPs, which proves the freshly prepared MR gels are isotropic and have good interfacial wettability between CIPs and the prepared PU matrix. The dynamic properties of prepared MR gels are obtained under LAOS. The influences of strain amplitude, applied coil current, CIPs weight fraction and driving angular frequency on the dynamic non-linear rheological properties of MR gels are systematic measured. It is found that the onset strain from linear viscoelastic region to non-linear viscoelastic region increases with increasing applied coil current and CIPs weight fraction. The maximum loss modulus value increases with increase of applied coil current and CIPs mass fraction. The larger the coil current and CIPs mass fraction, the greater the shear strain corresponding to the maximum value. The storage and loss modulus of the PU matrix does not vary and show current-independent characteristics. Polymer chains behaves like a linear sticky kettle with a broad LVE area since the loss modulus is much larger than storage modulus. Loss factor exhibits three characteristic regions (flat, up and drop) in the absence of coil current. However, there are four various domains of loss factor (flat, up, flat, up) presenting under the action of current. The dynamic non-linear respond of as-prepared samples is intensely related to the capacity for rearrangement of CIPs under the presentence of applied coil current. The magnetic Payne effect rely intensely on applied coil current and CIPs mass fraction. Under the cycle strain sweep, the approximate shape of the curve also remains unchanged. It seems that the magnetic-induced CIP chains rearrangement inside the MR gels is not effected by the inverse shear strain deformation even in the non-LVE area. Under the dynamic test of angular frequency sweep, the loading angular frequency has little impact on the dynamic rheological properties of self-developed MR gels, neither for the different applied coil current nor for the sample with different CIPs mass fraction.

## Conflicts of interest

There are no conflicts to declare.

## Supplementary Material
